# Opportunities and Challenges for Genetic Studies of End-Stage Renal Disease in Canada

**DOI:** 10.1177/2054358118789368

**Published:** 2018-07-22

**Authors:** Vinusha Kalatharan, Mathieu Lemaire, Matthew B. Lanktree

**Affiliations:** 1 Department of Epidemiology & Biostatistics, Western University, London, ON, Canada; 2 Division of Nephrology, The Hospital for Sick Children, University of Toronto, ON, Canada; 3 Cell Biology Program, SickKids Research Institute, Toronto, ON, Canada; 4 Division of Nephrology, University Health Network, Toronto, ON, Canada; 5 University of Toronto, ON, Canada; 6 Division of Nephrology, McMaster University, Hamilton, ON, Canada

**Keywords:** genetics, administrative data, ESRD, biobanks, precision medicine, chronic kidney disease

## Abstract

**Purpose of review:**

Genetic testing can improve diagnostic precision in some patients with end-stage renal disease (ESRD) providing the potential for targeted therapy and improved patient outcomes. We sought to describe the genetic architecture of ESRD and Canadian data sources available for further genetic investigation into ESRD.

**Sources of information:**

We performed PubMed searches of English, peer-reviewed articles using keywords “chronic kidney disease,” “ESRD,” “genetics,” “sequencing,” and “administrative databases,” and searched for nephrology-related Mendelian diseases on the Online Mendelian Inheritance in Man database.

**Methods:**

In this narrative review, we discuss our evolving understanding of the genetic architecture of kidney disease and ESRD, the risks and benefits of using genetic data to help diagnose and manage patients with ESRD, existing public Canadian biobanks and databases, and a vision for future genetic studies of ESRD in Canada.

**Key findings:**

ESRD has a polygenic architecture including rare Mendelian mutations and common small effect genetic polymorphism contributors. Genetic testing will improve diagnostic accuracy and contribute to a precision medicine approach in nephrology. However, the risk and benefits of genetic testing needs to be considered from an individual and societal perspective, and further research is required. Merging existing health data, linking biobanks and administrative databases, and forming Canadian collaborations hold great potential for genetic research into ESRD. Large sample sizes are necessary to perform the suitably powered investigations required to bring this vision to reality.

**Limitations:**

This is a narrative review of the literature discussing future directions and opportunities. It reflects the views and academic biases of the authors.

**Implications:**

National collaborations will be required to obtain sample sizes required for impactful, robust research. Merging established datasets may be one approach to obtain adequate samples. Patient education and engagement will improve the value of knowledge gained.

## Abrégé

**Contexte justifiant l’étude:**

Le dépistage génétique a le potentiel d’améliorer la précision diagnostique chez certains patients atteints d’insuffisance rénale terminale (IRT), et ouvre la voie à des thérapies ciblées et à de meilleures perspectives de santé pour les patients. Notre objectif est de présenter l’architecture génétique de l’IRT ainsi que les sources de données disponibles au Canada propres à faire avancer la recherche génétique sur l’IRT.

**Sources:**

Nous avons répertorié des articles rédigés en anglais, révisés par les pairs et publiés sur PubMed à l’aide des mots-clés suivants : *chronic kidney disease* (insuffisance rénale chronique), *ESRD* (IRT), *genetics* (génétique), *sequencing* (séquençage) et *administrative databases* (bases de données administratives). Nous avons également consulté la base de données OMIM (*Online Mendelian Inheritance in Man*) à la recherche de maladies néphrologiques mendéliennes.

**Méthodologie:**

Plusieurs aspects sont abordés dans le présent article synthèse : i) l’évolution de notre compréhension de l’architecture génétique de la maladie rénale et de l’IRT; ii) les risques et avantages de recourir aux données génétiques pour faciliter le diagnostic et la prise en charge des patients atteints d’IRT; iii) un répertoire des biobanques et bases de données publiques canadiennes, et iv) une vision pour la recherche en génétique sur l’IRT au Canada.

**Constats:**

L’IRT présente une architecture polygénique qui comprend de rares mutations mendéliennes et des contributeurs communs de polymorphisme génétique à effets mineurs. Le dépistage génétique contribuera à la précision du diagnostic et à l’adoption d’une médecine de précision en néphrologie. Cependant, les risques et avantages du dépistage génétique doivent être mesurés dans une perspective individuelle et sociétale, et des analyses supplémentaires s’imposent. La colligation des données en santé, des biobanques et des bases de données administratives existantes, couplée à des collaborations nationales, offre un vaste potentiel en recherche génétique sur l’IRT; il reste que des échantillons plus volumineux sont nécessaires pour étayer adéquatement les études qui permettront de concrétiser ce potentiel de recherche.

**Limites:**

Il s’agit d’un article synthèse de la documentation existante sur les perspectives et orientations d’avenir du point de vue des auteurs; l’article reflète conséquemment leur opinion et parti pris.

**Conclusion:**

Il nous faudra établir des collaborations nationales pour composer des échantillons dont la taille permettra de mener des recherches fructueuses et fiables. La colligation des jeux de données existants pourrait s’avérer une première approche à adopter pour y arriver. En outre, l’éducation des patients et leur participation à la recherche bonifieront la valeur des connaissances acquises.

## Why is this review important?

International efforts to develop large biobanks of the general population are underway. Canada’s ethnic diversity and unique populations are opportunities for furthering our understanding of genetics of kidney disease. Patients, clinicians, and researchers will need to work together to translate genetic research into advances in clinical practice.

## What are the key messages?

Generation of large collaborative biobanks is required to advance our understanding of the genetic underpinnings of chronic and end-stage kidney disease. Where possible, merging of existing administrative and clinical records is one mechanism to utilize existing data. Opportunity exists for Canada to unite efforts, leading to new discoveries in the genetic basis of kidney disease.

## Introduction

**Figure 1.**
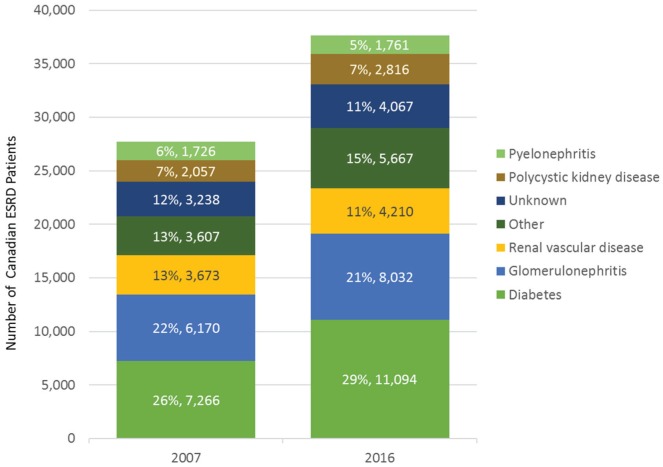
Stacked bar chart of prevalent ESRD cases in Canada (excluding Quebec) according to Canadian Institute for Health Information data (www.cihi.ca). *Note.* Diagnosis often cannot be made with a definitive test; thus, the validity of diagnosis may be unreliable. Moreover, disease progression is often multifactorial. Mendelian disorders likely account for >10% of ESRD, and a yet unknown and underappreciated polygenic component requires further study. Furthermore, genetic investigations may shed light onto the 11% of cases with unknown etiology. “Other” includes 29 conditions including drug-induced nephropathy, Alport syndrome, Fabry disease, oxalosis, cystinosis, Drash syndrome, HIV nephropathy, sickle cell nephropathy, multiple myeloma, amyloidosis, tuberculosis, gout, and Balkan nephropathy, among others. ESRD = end-stage renal disease.

**Figure 2.**
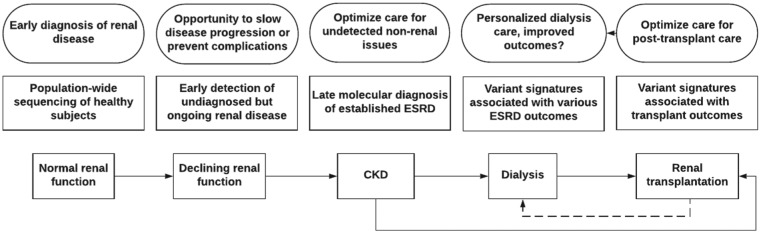
Illustration of the various steps where in-depth genetic analysis may help improve outcomes during the odyssey of a typical patient diagnosed with adult-onset ESRD. *Note.* ESRD = end-stage renal disease; CKD = chronic kidney disease.

Chronic kidney disease (CKD) is a persistent structural or functional abnormality of the kidneys. Over 2.9 million Canadians have CKD, and more than 35 000 have progressed to end-stage renal disease (ESRD).^1^ Our ability to successfully identify and treat patients with CKD with the greatest risk of progression to ESRD remains largely limited. ESRD is a heterogeneous outcome resulting from numerous potentially overlapping etiologies and pathophysiologic disease processes, such as hypertension, diabetic nephropathy, interstitial fibrosis, glomerulonephritis, or congenital anomalies of the urinary tract. The cause of ESRD is unknown in 11% of Canadian patients,^2^ while many more patients receive a clinical diagnosis without a definitive test (Figure 1). A precision medicine approach that includes traditional history, signs and symptoms, renal biopsy pathology, and biochemical biomarkers, as well as imaging and genetic investigations will contribute to our understanding of how and/or why a patient developed ESRD (Figure 2).^3,4^ Appropriate investigations in accordance with patient values and economic realities must be considered. Precision medicine may improve diagnostic clarity, prognostic accuracy, and therapy selection and provide valuable information for family planning. In this narrative review, we discuss the evolution of genetic investigations for patients with ESRD and the benefits and challenges of obtaining a genetic diagnosis. We highlight the unique opportunities available to study ESRD genomics through leverage of high-quality health data available in diverse Canadian patient populations.

## Methods

We sought to review current evidence for the architecture of chronic and end-stage kidney disease and the existing administrative and biobank resources available in Canada. We utilized PubMed searches of English, peer-reviewed articles using keywords “chronic kidney disease,” “ESRD,” “genetics,” “sequencing,” and “administrative databases” and searched for nephrology-related Mendelian diseases on the Online Mendelian Inheritance in Man database. Data tables were generated and information was synthesized into a narrative review. Internal and external peer review was performed as part of the KRESCENT training program.

## Review

### The Changing Landscape of Genetic Investigations

Advances in technology over the last decade have revolutionized the study of genetics in kidney diseases. Microarray technology allows rapid genotyping of hundreds of thousands of preselected common single nucleotide polymorphisms (SNPs) and was critical to the emergence of genome-wide association studies (GWAS).^5^ The development of high-throughput next-generation sequencing in turn enabled rapid genotyping of rare variants. Next-generation sequencing can now read gigabases (×109 base pairs) of sequence in a few hours for a few thousand dollars. Selection of the gene-containing region of the genome (exome sequencing)^6^ or targeted genes of interest (gene panels) improves efficiency and reduces cost.^7^ Molecular barcoding by ligating a short stretch of manufactured bases to each DNA sample allows for multiplexing many samples into one instrument run, further reducing cost and improving efficiency.

#### Evolving understanding of genetic kidney diseases

Large population-based GWAS identified thousands of associations between SNPs and quantitative phenotypes or diseases following a “common variant-common disease model” (Table 1).^8^ The largest genetic study in nephrology recruited >175 000 participants for a GWAS focused on serum creatinine and estimated glomerular filtration rate (eGFR). This study identified >60 strongly associated loci, yet they only explain a modest proportion of variability in the population (~2%-5%).^9,10^ These alleles are expected to each have small effect sizes because they are frequent in the general population (minor allele frequencies >5%) and have persisted despite natural selection. It is unclear how these results translate into concrete risk estimation for the development of ESRD because the majority of study participants had eGFR >60 mL/min/1.73 m_2_. Thus, genotyping these common SNPs currently provides minimal clinically relevant information to individual patients.

**Table 1.** Genetic Models of Complex Diseases Including Chronic Kidney Disease and End-Stage Renal Disease.

Model	Example diseases	Typical onset	No. of genes involved	Variant effect size	No. of patients affected	Predictive ability	Study design
Common variant-common disease	Hypertension, diabetes	Adulthood	Hundreds	Small	Many	Probabilistic	Genome-wide association studies
Rare variant-rare disease	Fabry disease, Alport syndrome	Pediatric to early adulthood	Single	Large	Few	Deterministic	Sequencing affected families, linkage analysis
Polygenic model	Chronic kidney disease	All	Hundreds	Small-to-large	All	Varies	Sequencing of large populations

In contrast, the “Rare Disease-Rare Variant model” describes classical Mendelian traits, where a large-effect mutation causes disease with high penetrance following an autosomal dominant, autosomal recessive, or X-linked inheritance pattern. Based on current estimates, 20% of CKD occurring in patients below the age of 25 years are due to a rare mutation from a growing list of genes (Table 2).^11^ Both locus heterogeneity (mutations in different genes produce the same disease) and allelic heterogeneity (different mutations in the same gene produce different phenotypes) are common. Generally, population-based GWAS have identified common SNPs with small effect sizes in genes that harbor mutations in Mendelian forms of disease (for example in height, body mass index, diabetes, or lipid levels).^12^ However, efforts to identify common variants affecting eGFR in genes that cause rare monogenic renal diseases have been largely unsuccessful, with a few exceptions (*UMOD, LRP2, SLC7A9*).^9,10,13^

**Table 2.** Growing List of Monogenic Diseases in Nephrology.

	Disease (Genes)
Tubular disease	Bartter syndrome (*SLC12A1, KCNJ1, CLCNKA, CLCNKB, BSND, CASR*) Gitelman syndrome (*SLC12A3*), Liddle syndrome (*SCNN1B, SCNN1G*) Cystinuria (*SLC3A1, SLC7A9)* Hyperoxaluria (*AGXT, GRHPR, HOGA1*) Renal glucosuria (*SGLT1, SGLT2*) Renal hypouricemia (*SLC22A12, SLC2A9)* Renal tubular acidosis (*SLC4A1, SLC4A4, ATP6V1B1, ATP6VOA1, ATP6VOA4, SLC34A1, CA2)* Rickets (*FGF23, DMP1, ENPP1, PHEX*) Pseudohypoaldosteronism, type 1 (*SCNN1A, NR3C2*) Pseudohypoaldosteronism, type 2 (*WNK1, WNK4, CUL3, KLHL3*) Hyperaldosteronism (*CYP11B1, CYP11B2, CACNA1D*) Apparent mineralocorticoid excess (*HSD11B2*) Nephrogenic diabetes insipidus (*AVPR2, AQP2)* Donnai-Barrow syndrome (*LRP2)*
Glomerular disease	Alport syndrome (*COL4A3, COL4A4, COL4A5, COL4A6, MYH9*) Steroid resistant nephrotic syndrome (*NPHS1, NPHS2, NPHS3-10: PLCE1*, ***WT1***, *LAMB2, PTPRO, DGKE, ARHGDIA, ADCK4, EMP2; EXT1, LMX1B, NUP93, NEU1, CUBN, ALG1, SMARCAL1*) Focal segmental glomerulosclerosis (*FSGS*1-9: *ACTN4, TRPC6, CD2AP, APOL1, INF2, MYO1E, PAZ2, ANLN, CRB2; LAMB2, LMNA, ARHGAP24, ITGB4, ITGA3, COL4A3-5, NXF5, NUP107, PDSS2, MTTL1, ZMPSTE24, PMM2, TTC21B, WDR73, FAT1)* Fabry disease (*GLA)* Nail patella syndrome (*LMX1B*) Glomerulopathy with fibronectin deposits (*FN1*)
Interstitial disease	Autosomal dominant tubulointerstitial disease (*UMOD, MUC1, REN*) Dent disease (*CLCN5, OCRL*) Mitochondrial complex III deficiency (*BCS1L, UQCRB, UQCRQ*) Senior-Loken syndrome (*CEP290*)
Thrombotic microangiopathy	Atypical hemolytic uremic syndrome, C3 glomerulopathy (*CFH, CFI, MCP, THBD, CFB, C3, CD46, ADAMTS13, DGKE*)
Structural disease	Congenital abnormalities of the kidney and urinary tract (*AGT, AGTR, BMP7, BMP4, CDC5L, CHD1L, DSTYK, ERCC4, EYA1, FGF20, FGFR1, FGFR2, FOXC1, FOXF1, FRAS1, FREM1, FREM2, GATA3, HNF1B, KAL1, MYOG, PAX2, RET, ROBO2, SALL1, SIX1, SIX2, SIX5, SLIT2, DLL3, DHCR7, PROK2, PROKR2, SOX9, SOX17, TNXB, TRAP1, UPK3A, WNT4*)
Cystic disease	Autosomal dominant polycystic kidney disease (*PKD1, PKD2)* Autosomal recessive polycystic kidney disease (*PKHD1, DZIP1L*) HNF1beta nephropathy (*HNF1B*) Hereditary angiopathy, neuropathy, aneurysms and muscle cramps (*COL4A1*) Hyperinsulinemia with hypoglycemia and polycystic kidney disease (*PMM2*) Nephronophthisis, Joubert, Meckel-Gruber syndrome (*NPH1-13, CEP290, ABCD3, MKS1, TMEM216, TMEM67, CCD2D2A*) Orofaciodigital syndrome (*OFD1*) **Neurofibromatosis (*NF1*)** **Tuberous sclerosis complex (*TSC1***, ***TSC2*)** **Von Hippel-Lindau (*VHL)***
Metabolic disease	Bardet-Biedl syndromes (*BBS1-15)* Coenzyme Q10 deficiency (*COQ2, APTX, PDSS2, CABC1, COQ9*) Fanconi anemia (*FANCA, FANCC, FANCD2, FANCE, FANCF, FANCG, FANCI, FANCL, FANCM, PALB2, BRIP1)* **Hereditary paraganglioma-pheochromocytoma syndrome (*SDHD***, ***SDHAF2***, ***SDHC***, ***SDHB*)** Hypoparathyroidism, sensorineural deafness, renal abnormalities (*GATA3*) Mitochondrial encephalomyopathy, lactic acidosis, and stroke-like episodes (*MTTL1*) **Multiple endocrine neoplasia (*MEN1***, ***RET*)** Nephrolithiasis (*SLC7A9, ADCY10, SLC2A9, SLC9A3R1, SLC22A12, SLC4A1, SLC3A1, ATP6V1B1, CLCN5, CLDN16, CYP24A1, AGXT, SLC34A1, SLC34A3, APRT*) **Wilson’s Disease (*ATP7A)***

*Note.* Mutations in genes indicated in bold script necessitate reporting as a secondary finding in clinical exome sequencing according to the American College of Medical Genetics and Genomics (ACMG).^14^

These two models cumulatively explain a portion of eGFR variability in the general population, and ESRD risk. However, this remains an oversimplification and a combined “polygenic model” is arising as the best explanation.^12^ A combination of both protective and deleterious common and rare variants are likely to contribute to the phenotypic expression of even classically defined Mendelian diseases. Digenic compound heterozygosity of deleterious alleles has been observed in polycystic kidney disease^15^ and Alport syndrome.^16^ Additional mechanisms may also contribute to population trait variability and penetrance, including somatic mosaicism, small noncoding or microRNA, epigenetic regulation, posttranslational modifications, variable X-inactivation, and gene-gene and gene-environment interactions. Finally, it is important to note that individual risk prediction is not directly tied to the proportion of explained variation in the population. For example, a mutation with a large effect on its carrier is likely rare enough to have minimal impact on population trait variability.^12^ A deeper understanding of the genetic basis of ESRD will require genetic studies to incorporate a polygenic model, requiring a large amount of data from a representative study population. Large samples will be possible by linking health care administrative databases and biobanks that are developing or in place across Canada.

### Genetic Testing in ESRD

While the list of Mendelian kidney diseases has grown significantly in recent years (Table 2), utilization of clinical genetic testing remains low among adult nephrologists. This may be due to 2 common misconceptions. The first misconception is that adult-onset ESRD is almost never due to a genetic mutation. The second misconception is that even if a mutation is discovered, it would be unlikely to alter patient management or disease outcome. Although the prevalence of each Mendelian disease is individually rare, the cumulative population prevalence of all Mendelian kidney diseases is surprisingly high.^17^ Strategies for prioritizing ESRD patients for genetic investigations include targeting those with early-onset disease (<45 years old), unclear or absent biopsy results, family history of any renal disease, or presence of extrarenal manifestations. After screening for genetic kidney disease risk factors, a multicenter study applied whole-exome sequencing to ~25% of more than 350 incident patients with CKD, with bona fide pathogenic mutations identified in 22 of 92 (24%) of these patients.^17^ Discovery of a genetic etiology of disease can have important ramifications, especially when considering disease recurrence risk posttransplantation. The purpose, consent process, sources of funding, and laboratory standards are different between clinical- and research-based genetic testing. However, they are not completely disparate entities, especially in the context of high throughput gene panel and exome genetic testing. If proper consent is obtained, DNA and genetic information collected for clinical genetic tests can form a rich resource for further genetic research. Conversely, samples collected for genetic research can lead to findings that could have clinical implications in a single participant. Ensuring the consent process is transparent and includes infrastructure and protocols for returning information to patients and research participants is of the utmost importance. Research-based genetic findings should be replicated in a Clinical Laboratory Improvement Amendments (CLIA)–certified laboratory to ensure veracity.

#### Risks and benefits of identification of a genetic cause of ESRD

Ideally, genetic testing would uncover the cause of CKD early in the course of the disease long before the development of ESRD. Genetic testing may uncover an underlying pathological mechanism, prevent unnecessary investigations including renal biopsy, shorten the diagnostic odyssey, improve risk prediction and stratification, and provide an opportunity for precision therapy. Identification of a mutation may also allow for identification and treatment of sometimes subtle extrarenal manifestations that may have been overlooked.^11^ Identification of a genetic cause of ESRD would also allow for low-cost cascade testing of family members, and opportunity for genetic counseling for family planning. It may also help estimate the risk of kidney disease recurrence after renal transplantation. Recognized and unrecognized negative consequences of genetic testing in ESRD also exist. When discussing the benefits of genetic testing with patients, it is important to also highlight some of the potential challenges that it inevitably triggers, such as dealing with incidental findings, risk of genetic discrimination, and interpretation of variants of uncertain significance.

#### Incidental genetic findings

High-throughput genetic testing, including whole-genome, exome, and gene panel sequencing, has led to issues regarding the reporting of incidental findings.^18^ Incidental findings include variants unrelated to the disease under scrutiny, but that could nevertheless be medically relevant to the patient or their extended family.^18^ The American College of Medical Genetics recommends a systematic check for pathogenic variants in 59 genes associated with 27 severe but treatable Mendelian conditions when performing clinical exome testing (the subset of the 59 genes relevant to nephrologists are in bold in Table 2).^14^ Variants in these genes are now defined as “secondary findings” as they must be actively sought out,^14^ and “incidental findings” encompass pathogenic variants found in other genes throughout the genome. In research settings, it is important to clarify if the patient wants to be made aware of secondary or incidental findings as they have the right to opt out.^19-22^ Secondary findings are common, as whole-genome sequencing data from 1000 healthy adults revealed that ~1% had a mutation in one of these genes.^23,24^ These issues must be taken into account when planning large-scale sequencing projects to minimize liability and budget for the additional costs triggered by these disclosures (eg, genetic counseling).^25,26^

#### Variants of uncertain significance

The American College of Medical Genetics provides guidelines to assess the pathogenic potential of rare missense variants. This exercise remains quite challenging, and many cases that remain inconclusive are labeled “variants of uncertain significance.” Comparison with large sequence databases is the first step, as >99% of causative rare variants have minor allele frequencies below 0.01%.^27^ Delineation of the co-segregation pattern of a rare variant with affection status in families can be helpful, but is often impractical or not possible. Bioinformatics tools can estimate a variant’s pathogenic potential, but unfortunately they frequently overestimate the pathogenicity of variants.^28^ Functional validation of variants using in vitro or in vivo models remains the gold standard, but it is very costly and time-consuming. At present, the pathogenicity of the vast majority of variants found during genetic testing is unclear.

#### Genetic discrimination in Canada and abroad

The acceptance and use of genetic testing in clinical and research settings may be dramatically hampered if genetic discrimination is not prevented. Genetic discrimination is defined as “adverse treatment that is based solely on the genotype of asymptomatic individuals.”^29^ For example, health insurers have declined to offer coverage for at-risk individuals who disclosed genetic information, including family history.^30^ There have also been cases where employers have used this information to dismiss potential or current employees.^31^ All members of European Union enacted legislation against genetic discrimination in the 1990s,^32^ as did the United States in 2008 with the passage of Genetic Information Nondiscrimination Act (GINA).^33,34^ Canada remained the only G7 country without clear genetic discrimination legislation until the Genetic Non-Discrimination Act became law in Canada in May 2017.^35,36^ This had real consequences as Canadians were routinely refused life and disability insurance on the basis genetic risk factors or family history.^37^ Similar problems have occurred in other developed nations with national health care systems, such as Japan^38^ and Australia.^39^ In contrast to European and American legislations, the Canadian Genetic Non-Discrimination Act adopted a narrower definition of “genetic information,” exclusively focusing on genetic testing results. Indeed, clinical information and family history are not protected under the Genetic Non-Discrimination Act even though these data provide information about the genetic makeup of an individual. While the Genetic Non-Discrimination Act is undeniably a positive development toward implementation of more widespread genetic testing in Canada, life and health insurers could use this loophole for lawful genetic discrimination. In sum, patients will need to weigh the potential risks and benefits from participation in genetic studies of ESRD.

### Linking Canadian Administrative Databases and Biobanks for Genetic Research

Canada’s universal and publicly funded health care system provides a wealth of administrative data to researchers. Each citizen has a unique health identifier, allowing for reliable data linkage across databases.^40^ Administrative data provide inexpensive access to large observational, population-based datasets with long follow-up times. The Canadian Institute for Health Information (CIHI) is a nonprofit organization that collects and analyzes these data and facilitates public access to health data (Table 3).^41^

**Table 3.** Description of Some Health Care Administrative Databases Maintained by the Canadian Institute for Health Information.

Database	Description
Discharge Abstract Database (DAD)	Contains administrative, demographic, and clinical information of all hospital discharges in all provinces and territories in Canada, except Quebec
National Ambulatory Care Reporting System (NACRS)	Contains demographic, administrative, and clinical information of all day surgeries, outpatient and community-based clinic visits, and emergency department visits
Continuing Care Reporting System (CCRS)	Contains complete or partial demographic, clinical, functional, and resource use information on all individuals receiving continuing care in hospital or long-term care homes. These data are available from Yukon, British Columbia, Alberta, Saskatchewan, Manitoba, Ontario, New Brunswick, Nova Scotia, and Newfoundland and Labrador
National Rehabilitation Reporting System (NRS)	Contains administrative, demographic, and clinical information from all adult inpatient rehabilitation facilities and programs across Canada
Canadian Organ Replacement Register (CORR)	Contains details pertaining to the type and outcomes of dialysis and transplantation

In parallel, investigators from across Canada have developed private biobanks which include genetic testing data. Canada’s largest prospective biobank initiative is the Canadian Partnership for Tomorrow Project (CPTP) that includes subjects from 5 cohorts recruited in 8 provinces (Table 4).^42^ The aim of CPTP is to identify environmental, lifestyle, and genetic risk factors of diseases, including CKD.^43^ The CPTP plans to continue to collect health information until at least the year 2033 and will ultimately include a myriad of data points for hundreds of thousands of Canadians.^42^

**Table 4.** Regional Cohorts of the Canadian Partnership for Tomorrow Project (CPTP).

Regional cohort	Provinces	Recruitment		No. of participants	No. of biosamples	Type of biosamples	Age (years)
		Start date	End date				
BC Generation Project	British Columbia	February 2008	February 2015	43,068	27 000	Blood Urine Saliva	40-69
Alberta’s Tomorrow Project	Alberta	January 2000	December 2015	55 000	30 000	Blood Urine Saliva	35-69
Ontario Health Study	Ontario	March 2009	March 2017	165 476	40 660	Blood Urine	>18
CARTaGENE	Quebec	February 2008	February 2015	43 068	30 283	Blood Urine Saliva	40-69
Atlantic Path	Nova Scotia, New Brunswick, Prince Edward Island, Newfoundland and Labrador	March 2009	December 2015	35 471	32 512	Blood Urine Saliva Toenail Clipping	18-78

Studies linking biobanks to administrative data can overcome the shortcomings of each type of data source. Existing biobanks generally lack the detailed health care utilization data available in administrative databases, and administrative databases lack biological information found in biobanks. It is important to acknowledge that administrative data are not collected with the same rigor used in research studies; therefore, the quality and reliability of the data can be affected.^44^ Researchers who use health care administrative data typically identify diseased patients and build study cohorts using the *International Classification of Disease, Ninth Revision* (*ICD-9*) or *Tenth Revision* (*ICD-10*) codes, or physician claim diagnosis codes. Although *ICD-9* and *ICD-10* codes are highly specific, they are not sensitive because they only identify patients with a hospital admission or emergency department visit who usually have a more advanced disease.^45,46^ In contrast, physician claim diagnosis codes generally have poorer specificity but greater sensitivity to capture a much higher percentage of the relevant population. Linking genetic information from biobanks to administrative databases would allow researchers to assemble study cohorts with definitive diagnoses and broader disease spectra. It would also provide invaluable phenotypic information for large-scale genotype-phenotype association studies.

#### Opportunities and barriers to merging health data across jurisdictions

Merging health data (including administrative data and biobanks) across provincial and/or territorial jurisdictions could increase research capacity in Canada. For example, merging databases allows for assembly of larger cohorts of patients that may include several individuals with rare conditions. Merging may also help avoid duplication of efforts, such as having multiple investigators collecting the same data to answer overlapping research questions. It could also increase researchers’ access to data from jurisdictions that are outside of their home province/territory. For example, the Ontario Drug Benefits dataset contains prescription drug claims limited to a small subset of the population that primarily consist of individuals over the age of 65 years. In contrast, every Québecers without private prescription drug insurance is covered under the public drug insurance plan (Régie de l’Assurance Maladie du Québec) and thus contains information from patients with a more diverse age range. Broad access to these 2 databases for Canadian researchers (especially if merged) would allow them to ask unique questions using the most relevant data available.

Unfortunately, several barriers prevent merging of provincial and territorial health care administrative data. The major barriers that currently exist and potential solutions to circumvent them are summarized in Table 5.^40,47,48^ A report published by CIHI in 2002 provides a detailed account of specific legislative barriers to data linkage across Canadian jurisdictions that is still relevant.^48^

**Table 5.** List of Few Barriers to Link Data Across Jurisdictions in Canada and Potential Approaches to Overcome the Barriers.

Barriers	Approach to overcome barrier
*Variation in legislation, policies, and privacy and confidentiality review protocols across provinces and territories in Canada*: Laws prevent administrative data transfer across jurisdictions and demand data linkage to occur by province by province to conduct national studies.^47^	Standardize laws, policies, and privacy and confidentiality review protocols across jurisdictions.
*Cost*: The price for linking data ranges $5000 to $90 000 per province.^40^	Canadians can gather, learn from each others’ systems, and identify ways to reduce cost and link data in a more cost efficient manner.
*Substantial difference in coding practices across jurisdiction in Canada*: Some provinces and territories, such as British Columbia, developed internal coding systems, while others, such as Ontario, adopted a standardized coding system.^48^	For now, researchers must rely on the National Grouping System developed by Canadian Institute for Health Information to convert coding practices in each jurisdiction to a standard one. However, standardization of coding practices across Canadian jurisdictions would be ideal.^48^

There are several initiatives to link biobanks to administrative databases in Canada. For example, the British Columbia (BC) Generation Project is linked to administrative Population Data BC on an ongoing basis.^49^ We should continue to populate existing biobanks, to establish new regional cohorts in jurisdictions not currently involved in CPTP, and continue linking biobanks with administrative databases. The success of the continual expansion of biobanks and linkage to administrative databases for genetic research is highly reliant on Canadians’ willingness to share their detailed medical and genetic information. Engaging patients can help with this and with genetic research in general.

### Patient Engagement to Facilitate Proliferation of Genetic Research in Canada

Patient engagement occurs when researchers, patients, and caregivers collaborate “in the governance, priority setting, and conduct of research, as well as in summarizing, distributing, sharing, and applying its resulting knowledge” (ie, knowledge translation).^50^ Studies show that patient engagement leads to higher enrollment and retention rates and dissemination of findings in a more meaningful and understandable way.^51^ The Canadians Seeking Solutions and Innovation to Overcome CKD (Can-SOLVE CKD) network’s experience also emphasizes the value of patient engagement.^52^ The Can-SOLVE CKD network is a unique partnership between patients, researchers, practitioners, and policy makers to revolutionize the care of Canadians with CKD and is one of 7 networks funded under the Canadian Institutes for Health Research Strategy for Patient-Oriented Research initiative. Patient engagement lead to identification of top patient-relevant research priorities that created the foundations for the 18 research projects covered under the umbrella of Can-SOLVE CKD.^52^

Patient engagement is of particular importance in genetic research as it relies on patients’ willingness to share medical and genetic data. Assembling biobanks and linking them to health care administrative databases requires broad participant consent for use of both biological specimens and data for research. Patient collaborators are often willing to participate in all stages of the research process including setting research priorities, study design, research dissemination, and knowledge translation. They may provide insight into reasons that could deter others from participation and advise on methods to communicate incidental findings and how to communicate the issue with prospective participants during patient recruitment. Patient collaborators can help draft and revise consent forms to make them understandable to future participants and help communicate the importance of future genetic research when recruiting new patients. In addition, patient collaborators can share their experiential knowledge. Finally, they may help advocate to improve the Canadian Genetic Non-Discrimination Act. Patient engagement is essential for the development of robust genetic studies focused on ESRD.

### Avenues for Future Genetic Study of ESRD

Enrollment of large samples of patients in biobanks with linked administrative health data will provide the opportunity for study designs that include both common and rare genetic variants for the evaluation of both common quantitative traits and rare Mendelian diseases. Should investigators despair that sample sizes of hundreds of thousands of participants are required for novel genetic discoveries? Using extreme phenotypes, especially unaccounted for by traditional risk factors, is one strategy to increase power.^53^ For example, one could compare patients with diabetic nephropathy who develop early ESRD despite excellent risk factor management to those who progress slowly despite the presence of such risk factors. Furthermore, knowledge of a genetic association with phenotypes in the general population can be leveraged in the ESRD population. For example, variants associated with elevated fasting blood sugar or hemoglobin A1C in the general population could be tested for association with risk of developing new onset diabetes after kidney transplantation.

Canada’s ethnic diversity imposes both challenges and opportunities for genetic studies of ESRD. Databases of genetic data from control subjects are heavily focused on European and African American subjects limiting interpretation of rare variants in other populations. However, the multiethnic Canadian landscape provides a unique opportunity for transethnic and admixture association and mapping studies.^54^ Efforts to collate patients into extended pedigrees in databases will greatly improve power for studying rare variants in ESRD. Collaboration between centers to facilitate collection of patients into consortia creating the largest possible sample sizes is a necessity.

Research efforts using next-generation sequencing, uncovering Mendelian forms of CKD in ESRD, are currently of great interest. In contrast, do small effect common genetic variants even matter? Interest in GWAS has waned after the proliferation of next-generation sequencing, but many questions amenable to GWAS remain in nephrology. GWAS-derived common variants can provide insight into biology and potential therapeutic targets, but pinpointing the responsible genes in GWAS-identified regions is challenging and ongoing. Identified genes can be used as drug targets for creating larger effects than the observed effect of the SNP in the GWAS. GWAS-derived SNPs can be combined into polygenic risk scores, and patients identified with a rare collection of many common risk variants may present with a phenotype similar to those with Mendelian disease. Mendelian randomization studies jointly test the association between genetic variants and both a risk factor and an outcome to support that the risk factor is a causative contributor to the outcome. For example, reduced high-density lipoprotein (HDL) cholesterol appears a risk factor for CKD, as genetic variants associated with reduced HDL are also associated with CKD risk.^55^ However, Mendelian randomization requires GWAS-identified genetic predictors of the risk factor. In sum, incorporating both common small effect and rare large effect variants, as well as other sources of “missing heritability,” will be required to maximize the insight gained from genetic studies in ESRD.

#### Using genetic variants to optimize renal replacement therapy

The potential contribution of genetics to hemodialysis and peritoneal dialysis (PD) outcomes remains inadequately studied. Evidence is limited to candidate gene association studies, which have significant issues with reproducibility and publication bias. Studies looked for SNP associations with hemodialysis outcomes including vascular access issues,^56-61^ biochemical indices,^62-65^ inflammation,^66-68^ cardiovascular comorbidities^66,69-75^ and mortality^76-79^ and PD outcomes including peritoneal transport characteristics,^80-84^ peritonitis risk,^85-87^ or risk of encapsulating peritoneal sclerosis^88^ (Supplemental Table 1). Sample sizes were inadequate to confidently identify variants of reasonable effect sizes (average sample size of 246, range 67-777 patients). Only a single published GWAS relating to hemodialysis exists, published in 2011, examining survival in 647 African American ESRD patients with type 2 diabetes.^89^ No replication study has been published, nor are additional studies reportedly underway. Two ongoing studies could impart knowledge on PD. The first, PD-CRAFT (NCT02042768), is collecting clinical and genetic data on 1495 PD patients. The second, BIO-PD (Biological Determinants of Peritoneal Dialysis; NCT02694068), has an estimated enrollment of 4865 patients between 2014 and 2019. Both studies are part of the Peritoneal Dialysis Outcomes and Practice Patterns Study (PDOPPS), an international consortium formed to promote research aimed at improving practice and outcomes in PD.^90^ Genetic studies could improve our knowledge of renal replacement therapy pathophysiology and hold the potential for improving ESRD therapy.

### Looking Forward

Genetics could reduce diagnostic ambiguity and contribute to a precision medicine approach to ESRD care. Our understanding of the genetic architecture of complex diseases is growing rapidly, and these advances are applicable to ESRD. Canadians should build a rich data repository for health research, linking and merging health data including biobanks and administrative and clinical databases, across jurisdictions. To facilitate the proliferation of large-scale genetic studies in Canada, it is imperative to overcome the barriers to data linkage by standardizing coding practices, legislation, policies, ethics, privacy and confidentiality review protocols, and genetic testing. Canadians should be protected by broadening genetic nondiscrimination legislation. It is important for patient representatives to be actively involved in setting the priorities for CKD genetic research because they are the ones that stand to benefit the most from this research. Large sample sizes will be required to draw robust conclusions. Artificial intelligence and machine learning algorithms could be applied to such data sources. Systemic changes are needed to ensure equitable attribution of credit to those who build, contribute, analyze, publish, or disseminate knowledge generated from datasets derived from linked databases. Building common biobank and database resources for improved study designs should be embraced by the Canadian nephrology community.

## Supplemental Material

Supplemental_table_1 – Supplemental material for Opportunities and Challenges for Genetic Studies of End-Stage Renal Disease in Canada Click here for additional data file. Supplemental material, Supplemental_table_1 for Opportunities and Challenges for Genetic Studies of End-Stage Renal Disease in Canada by Vinusha Kalatharan, Mathieu Lemaire and Matthew B. Lanktree in Canadian Journal of Kidney Health and Disease

## References

[1] Arora P Vasa P Brenner D et al Prevalence estimates of chronic kidney disease in Canada: results of a nationally representative survey. CMAJ. 2013;185:E417-E423. 23649413 10.1503/cmaj.120833 PMC3680588

[2] Canadian Institute for Health Information. Incident End-Stage Kidney Disease Patients: CORR data. https://www.cihi.ca/en/access-data-and-reports.

[3] Lanktree MB Chapman AB. New treatment paradigms for ADPKD: moving towards precision medicine. Nat Rev Nephrol. 2017;13:750-768. 28989174 10.1038/nrneph.2017.127

[4] Bierzynska A McCarthy HJ Soderquest K et al Genomic and clinical profiling of a national nephrotic syndrome cohort advocates a precision medicine approach to disease management. Kidney Int. 2017;91:937-947. 28117080 10.1016/j.kint.2016.10.013

[5] Wellcome Trust Case Control Consortium. Genome-wide association study of 14,000 cases of seven common diseases and 3,000 shared controls. Nature. 2007;447:661-678. 17554300 10.1038/nature05911 PMC2719288

[6] Warejko JK Tan W Daga A et al Whole exome sequencing of patients with steroid-resistant nephrotic syndrome. Clin J Am Soc Nephrol. 2017;13(1):53-62. 29127259 10.2215/CJN.04120417 PMC5753307

[7] Mori T Hosomichi K Chiga M et al Comprehensive genetic testing approach for major inherited kidney diseases, using next-generation sequencing with a custom panel. Clin Exp Nephrol. 2017;21:63-75. 26920127 10.1007/s10157-016-1252-1

[8] Schork NJ Murray SS Frazer KA Topol EJ. Common vs. rare allele hypotheses for complex diseases. Curr Opin Genet Dev. 2009;19:212-219. 19481926 10.1016/j.gde.2009.04.010 PMC2914559

[9] Pattaro C Teumer A Gorski M et al Genetic associations at 53 loci highlight cell types and biological pathways relevant for kidney function. Nat Commun. 2016;7:10023. 26831199 10.1038/ncomms10023 PMC4735748

[10] Gorski M van der Most PJ Teumer A et al 1000 Genomes-based meta-analysis identifies 10 novel loci for kidney function. Sci Rep. 2017;7:45040. 28452372 10.1038/srep45040 PMC5408227

[11] Vivante A Hildebrandt F. Exploring the genetic basis of early-onset chronic kidney disease. Nat Rev Nephrol. 2016;12:133-146. 26750453 10.1038/nrneph.2015.205 PMC5202482

[12] Timpson NJ Greenwood CMT Soranzo N Lawson DJ Richards JB. Genetic architecture: the shape of the genetic contribution to human traits and disease. Nat Rev Genet. 2018;19(2):110-124. 29225335 10.1038/nrg.2017.101

[13] Parsa A Fuchsberger C Köttgen A et al Common variants in Mendelian kidney disease genes and their association with renal function. J Am Soc Nephrol. 2013;24:2105-2117. 24029420 10.1681/ASN.2012100983 PMC3839542

[14] Kalia SS Adelman K Bale SJ et al Recommendations for reporting of secondary findings in clinical exome and genome sequencing, 2016 update (ACMG SF v2.0): a policy statement of the American College of Medical Genetics and Genomics. Genet Med. 2017;19:249-255. 27854360 10.1038/gim.2016.190

[15] Pei Y Paterson AD Wang KR et al Bilineal disease and trans-heterozygotes in autosomal dominant polycystic kidney disease. Am J Hum Genet. 2001;68:355-363. 11156533 10.1086/318188 PMC1235269

[16] Mohammad M Nanra R Colville D et al A female with X-linked Alport syndrome and compound heterozygous COL4A5 mutations. Pediatr Nephrol. 2014;29:481-485. 24337245 10.1007/s00467-013-2682-6

[17] Lata S Marasa M Li Y et al Whole-exome sequencing in adults with chronic kidney disease: a pilot study. Ann Intern Med. 2018;168(2):100-109. 29204651 10.7326/M17-1319 PMC5947852

[18] Green RC Berg JS Grody WW et al ACMG recommendations for reporting of incidental findings in clinical exome and genome sequencing. Genet Med. 2013;15:565-574. 23788249 10.1038/gim.2013.73 PMC3727274

[19] ACMG Board of Directors. ACMG policy statement: updated recommendations regarding analysis and reporting of secondary findings in clinical genome-scale sequencing. Genet Med. 2015;17:68-69. 25356965 10.1038/gim.2014.151

[20] Scheuner MT Peredo J Benkendorf J et al Reporting genomic secondary findings: ACMG members weigh in. Genet Med. 2015;17:27-35. 25394173 10.1038/gim.2014.165

[21] Wolf SM Crock BN Van Ness B et al Managing incidental findings and research results in genomic research involving biobanks and archived data sets. Genet Med. 2012;14:361-384. 22436882 10.1038/gim.2012.23 PMC3597341

[22] Wolf SM. The past, present, and future of the debate over return of research results and incidental findings. Genet Med. 2012;14:355-357. 22481182 10.1038/gim.2012.26 PMC4469992

[23] Dorschner MO Amendola LM Turner EH et al Actionable, pathogenic incidental findings in 1,000 participants’ exomes. Am J Hum Genet. 2013;93:631-640. 24055113 10.1016/j.ajhg.2013.08.006 PMC3791261

[24] Olfson E Cottrell CE Davidson NO et al Identification of medically actionable secondary findings in the 1000 genomes. PLoS One. 2015;10:e0135193. 26332594 10.1371/journal.pone.0135193 PMC4558085

[25] Evans BJ. Minimizing liability risks under the ACMG recommendations for reporting incidental findings in clinical exome and genome sequencing. Genet Med. 2013;15:915-920. 24030435 10.1038/gim.2013.135 PMC3892767

[26] Bennette CS Gallego CJ Burke W Jarvik GP Veenstra DL. The cost-effectiveness of returning incidental findings from next-generation genomic sequencing. Genet Med. 2015; 17:587-595. 25394171 10.1038/gim.2014.156 PMC4430464

[27] Kobayashi Y Yang S Nykamp K Garcia J Lincoln SE Topper SE. Pathogenic variant burden in the ExAC database: an empirical approach to evaluating population data for clinical variant interpretation. Genome Med. 2017;9:13. 28166811 10.1186/s13073-017-0403-7 PMC5295186

[28] Andersen LL Terczyńska-Dyla E Mørk N et al Frequently used bioinformatics tools overestimate the damaging effect of allelic variants [published online ahead of print December 4, 2017]. Genes Immun. doi:10.1038/s41435-017-0002-z. 29217828

[29] Rothstein MA Anderlik MR. What is genetic discrimination, and when and how can it be prevented? Genet Med. 2001;3:354-358. 11545689 10.1097/00125817-200109000-00005

[30] Hudson KL Rothenberg KH Andrews LB Kahn MJ Collins FS. Genetic discrimination and health insurance: an urgent need for reform. Science. 1995;270:391-393. 7569991 10.1126/science.270.5235.391

[31] Rothenberg K Fuller B Rothstein M et al Genetic information and the workplace: legislative approaches and policy changes. Science. 1997;275:1755-1757. 9122681 10.1126/science.275.5307.1755

[32] Van Hoyweghen I Horstman K European practices of genetic information and insurance: lessons for the Genetic Information Nondiscrimination Act. JAMA. 2008;300:326-327. 18632546 10.1001/jama.2008.62

[33] Hudson KL Holohan MK Collins FS. Keeping pace with the times—the Genetic Information Nondiscrimination Act of 2008. N Engl J Med. 2008;358:2661-2663. 18565857 10.1056/NEJMp0803964

[34] Soini S Genetic testing legislation in Western Europe—a fluctuating regulatory target. J Community Genet. 2012;3:143-153. 10.1007/s12687-012-0078-0 PMC3312949 22287154

[35] Statutes of Canada 2017 An Act to Prohibit and Prevent Genetic Discrimination. http://www.parl.ca/Content/Bills/421/Private/S-201/S-201_4/S-201_4.PDF. Accessed January 31, 2018.

[36] Joly Y Feze IN Song L Knoppers BM. Comparative approaches to genetic discrimination: chasing shadows? Trends Genet. 2017;33:299-302. 28365141 10.1016/j.tig.2017.02.002

[37] Bombard Y Veenstra G Friedman JM et al Perceptions of genetic discrimination among people at risk for Huntington’s disease: a cross sectional survey. BMJ. 2009;338:b2175. 19509425 10.1136/bmj.b2175 PMC2694258

[38] Murashige N Tanimoto T Kusumi E. Fear of genetic discrimination in Japan. Lancet. 2012;380:730. 22920751 10.1016/S0140-6736(12)61407-X

[39] Taylor S Treloar S Barlow-Stewart K Stranger M Otlowski M. Investigating genetic discrimination in Australia: a large-scale survey of clinical genetics clients. Clin Genet. 2008;74:20-30. 18492091 10.1111/j.1399-0004.2008.01016.x

[40] Quan H Smith M Bartlett-Esquilant G et al Mining administrative health databases to advance medical science: geographical considerations and untapped potential in Canada. Can J Cardiol. 2012;28:152-154. 22301469 10.1016/j.cjca.2012.01.005

[41] Canadian Institute for Health Information. https://www.cihi.ca/en. Accessed 31 January, 2018.

[42] Borugian MJ Robson P Fortier I et al The Canadian partnership for tomorrow project: building a pan-Canadian research platform for disease prevention. CMAJ. 2010;182:1197-1201. 20421354 10.1503/cmaj.091540 PMC2917932

[43] Canadian Partnership for Tomorrow Project. Date unknown. http://www.partnershipfortomorrow.ca/about/.Accessed 31, January 2018.

[44] Quan H Parsons GA Ghali WA. Validity of procedure codes in International Classification of Diseases, 9th revision, clinical modification administrative data. Med Care. 2004;42:801-809. 15258482 10.1097/01.mlr.0000132391.59713.0d

[45] Fleet JL Shariff SZ Gandhi S Weir MA Jain AK Garg AX. Validity of the International Classification of Diseases 10th revision code for hyperkalaemia in elderly patients at presentation to an emergency department and at hospital admission. BMJ Open. 2012;2:e002011. 10.1136/bmjopen-2012-002011 PMC4399109 23274674

[46] Molnar AO van Walraven C McArthur E Fergusson D Garg AX Knoll G. Validation of administrative database codes for acute kidney injury in kidney transplant recipients. Can J Kidney Health Dis. 2016;3:18. 27057318 10.1186/s40697-016-0108-7 PMC4823855

[47] Doiron D Raina P Fortier I. Linking Canadian population health data: maximizing the potential of cohort and administrative data. Can J Public Health. 2013;104:e258-e261. 23823892 10.17269/cjph.104.3775 PMC3880355

[48] Canadian Institute for Health Information. Barriers to Accessing & Analyzing Health Information in Canada. 2002 https://secure.cihi.ca/estore/productSeries.htm?pc=PCC174. Accessed 31 January, 2018.

[49] BC Generations Project Details. https://www.bcgenerationsproject.ca/about/study-details/. Accessed 31 January, 2018.

[50] Government of Canada. Patient Engagement. http://www.cihr-irsc.gc.ca/e/45851.html. 2012 Accessed February 26, 2018.

[51] Domecq JP Prutsky G Elraiyah T et al Patient engagement in research: a systematic review. BMC Health Serv Res. 2014;14:89. 24568690 10.1186/1472-6963-14-89 PMC3938901

[52] Levin A Adams E Barrett BJ et al Canadians Seeking Solutions and Innovations to Overcome Chronic Kidney Disease (Can-SOLVE CKD): form and function. Can J Kidney Health. 2018;5:2054358117749530. 10.1177/2054358117749530 PMC5774731 29372064

[53] Lanktree MB Hegele RA Schork NJ Spence JD. Extremes of unexplained variation as a phenotype: an efficient approach for genome-wide association studies of cardiovascular disease. Circ Cardiovasc Genet. 2010;3:215-221. 20407100 10.1161/CIRCGENETICS.109.934505 PMC3084495

[54] Li YR Keating BJ. Trans-ethnic genome-wide association studies: advantages and challenges of mapping in diverse populations. Genome Med. 2014;6:91. 25473427 10.1186/s13073-014-0091-5 PMC4254423

[55] Lanktree MB Thériault S Walsh M Paré G. HDL cholesterol, LDL cholesterol, and triglycerides as risk factors for CKD: a Mendelian Randomization Study. Am J Kidney Dis. 2018;7:166-172. 10.1053/j.ajkd.2017.06.011 28754456

[56] Ram S Bass K Abreo K Baier RJ Kruger TE. Tumor necrosis factor-alpha–308 gene polymorphism is associated with synthetic hemodialysis graft failure. J Investig Med. 2003;51(1):19-26. 10.2310/6650.2003.33522 12580317

[57] Lazo-Langner A Knoll GA Wells PS Carson N Rodger MA. The risk of dialysis access thrombosis is related to the transforming growth factor-beta1 production haplotype and is modified by polymorphisms in the plasminogen activator inhibitor-type 1 gene. Blood. 2006;108:4052-4058. 16931622 10.1182/blood-2006-06-028902

[58] Sener EF Taheri S Korkmaz K et al Association of TNF-α -308 G > A and ACE I/D gene polymorphisms in hemodialysis patients with arteriovenous fistula thrombosis. Int Urol Nephrol. 2014;46:1419-1425. 24126814 10.1007/s11255-013-0580-2

[59] Heine GH Ulrich C Sester U Sester M Köhler H Girndt M. Transforming growth factor beta1 genotype polymorphisms determine AV fistula patency in hemodialysis patients. Kidney Int. 2003;64:1101-1107. 12911563 10.1046/j.1523-1755.2003.00176.x

[60] Lin CC Yang WC Chung MY Lee PC. Functional polymorphisms in matrix metalloproteinases-1, -3, -9 are associated with arteriovenous fistula patency in hemodialysis patients. Clin J Am Soc Nephrol. 2010;5:1805-1814. 20616161 10.2215/CJN.01500210 PMC2974381

[61] Brophy DF Bukaveckas BL Ferreira-Gonzalez A Archer KJ Martin EJ Gehr TW. A pilot study of genetic polymorphisms and hemodialysis vascular access thrombosis. Hemodial Int. 2009;13:19-26. 19210273 10.1111/j.1542-4758.2009.00334.x

[62] Jeong KH Lee TW Ihm CG Lee SH Moon JY. Polymorphisms in two genes, IL-1B and ACE, are associated with erythropoietin resistance in Korean patients on maintenance hemodialysis. Exp Mol Med. 2008;40:161-166. 18446054 10.3858/emm.2008.40.2.161 PMC2679299

[63] Marchelek-Myśliwiec M Różański J Ogrodowczyk A et al The association of the Klotho polymorphism rs9536314 with parameters of calcium-phosphate metabolism in patients on long-term hemodialysis. Ren Fail. 2016;38:776-780. 27055909 10.3109/0886022X.2016.1162062

[64] Gohda T Shou I Fukui M et al Parathyroid hormone gene polymorphism and secondary hyperparathyroidism in hemodialysis patients. Am J Kidney Dis. 2002;39:1255-1260. 12046039 10.1053/ajkd.2002.33399

[65] Amato M Pacini S Aterini S Punzi T Gulisano M Ruggiero M. Iron indices and vitamin D receptor polymorphisms in hemodialysis patients. Adv Chronic Kidney Dis. 2008;15:186-190. 18334245 10.1053/j.ackd.2008.01.013

[66] Liu Y Berthier-Schaad Y Fallin MD et al IL-6 haplotypes, inflammation, and risk for cardiovascular disease in a multiethnic dialysis cohort. J Am Soc Nephrol. 2006;17:863-870. 16467451 10.1681/ASN.2005050465

[67] Girndt M Sester U Sester M et al The interleukin-10 promoter genotype determines clinical immune function in hemodialysis patients. Kidney Int. 2001;60:2385-2391. 11737614 10.1046/j.1523-1755.2001.00062.x

[68] Biolo G Amoroso A Savoldi S et al Association of interferon-gamma +874A polymorphism with reduced long-term inflammatory response in haemodialysis patients. Nephrol Dial Transplant. 2006;21:1317-1322. 16410273 10.1093/ndt/gfk033

[69] Asakimori Y Yorioka N Tanaka J et al Association between ENOS gene polymorphism and cardiovascular events in nondiabetic hemodialysis patients: a prospective study. Am J Kidney Dis. 2004;44:112-120. 15211444 10.1053/j.ajkd.2004.03.034

[70] Losito A Kalidas K Santoni S Ceccarelli L Jeffery S. Polymorphism of renin-angiotensin system genes in dialysis patients—association with cerebrovascular disease. Nephrol Dial Transplant. 2002;17:2184-2188. 12454231 10.1093/ndt/17.12.2184

[71] Losito A Kalidas K Santoni S Jeffery S. Association of interleukin-6 -174G/C promoter polymorphism with hypertension and left ventricular hypertrophy in dialysis patients. Kidney Int. 2003;64:616-622. 12846758 10.1046/j.1523-1755.2003.00119.x

[72] Yilmaz R Altun B Ozer N Hazirolan T Turgan C. Impact of cytokine genotype on cardiovascular surrogate markers in hemodialysis patients. Ren Fail. 2010;32:806-816. 20662694 10.3109/0886022X.2010.494798

[73] Tosic Dragovic J Popovic J Djuric P et al Relative risk for cardiovascular morbidity in hemodialysis patients regarding gene polymorphism for IL-10, IL-6, and TNF. Can J Physiol Pharmacol. 2016;94:1106-1109. 27580171 10.1139/cjpp-2015-0569

[74] Zheng ZL Hwang Y-H Kim SK et al Genetic polymorphisms of hypoxia-inducible factor-1 alpha and cardiovascular disease in hemodialysis patients. Nephron Clin Pract. 2009;113:c104-c111. 19602906 10.1159/000228542

[75] Rao M Guo D Jaber BL et al Transforming growth factor-beta 1 gene polymorphisms and cardiovascular disease in hemodialysis patients. Kidney Int. 2004;66:419-427. 15200451 10.1111/j.1523-1755.2004.00748.x

[76] Böger CA Fischereder M Deinzer M et al RANTES gene polymorphisms predict all-cause and cardiac mortality in type 2 diabetes mellitus hemodialysis patients. Atherosclerosis. 2005;183:121-129. 15899487 10.1016/j.atherosclerosis.2005.03.006

[77] Marco MP Craver L Betriu A Fibla J Fernández E. Influence of vitamin D receptor gene polymorphisms on mortality risk in hemodialysis patients. Am J Kidney Dis. 2001;38:965-974. 11684548 10.1053/ajkd.2001.28582

[78] Grzegorzewska AE Świderska MK Mostowska A Warchoł W Jagodziński PP. Polymorphisms of T helper cell cytokine-associated genes and survival of hemodialysis patients—a prospective study. BMC Nephrol. 2017;18:165. 28525983 10.1186/s12882-017-0582-x PMC5437603

[79] Rothuizen TC Ocak G Verschuren JJW et al Candidate gene analysis of mortality in dialysis patients. PLoS One. 2015;10:e0143079. 26587841 10.1371/journal.pone.0143079 PMC4654483

[80] Szeto CC Poon P Szeto CY Wong TY Lai KB Li PK. Plasminogen activator inhibitor-1 4G/5G genetic polymorphism does not affect peritoneal transport characteristic. Am J Kidney Dis. 2002;39:1061-1067. 11979351 10.1053/ajkd.2002.32790

[81] Wong TY Szeto CC Szeto CY Lai KB Chow KM Li PK. Association of ENOS polymorphism with basal peritoneal membrane function in uremic patients. Am J Kidney Dis. 2003;42:781-786. 14520629 10.1016/s0272-6386(03)00855-2

[82] Szeto C-C Chow K-M Poon P Szeto CY Wong TY Li PK. Genetic polymorphism of VEGF: impact on longitudinal change of peritoneal transport and survival of peritoneal dialysis patients. Kidney Int. 2004;65:1947-1955. 15086939 10.1111/j.1523-1755.2004.00605.x

[83] Gillerot G Goffin E Michel C et al Genetic and clinical factors influence the baseline permeability of the peritoneal membrane. Kidney Int. 2005;67:2477-2487. 15882295 10.1111/j.1523-1755.2005.00357.x

[84] Ding L Shao X Cao L et al Possible role of IL-6 and TIE2 gene polymorphisms in predicting the initial high transport status in patients with peritoneal dialysis: an observational study. BMJ Open. 2016;6:e012967. 10.1136/bmjopen-2016-012967 PMC5093628 27798027

[85] Uchiyama K Naito K Tsuchida M et al Impact of a genetic polymorphism of the interleukin-1 receptor antagonist on technique survival in peritoneal dialysis patients. Blood Purif. 2005;23:450-458. 16244470 10.1159/000088988

[86] Lam MF Leung JCK Tang CCS et al Mannose binding lectin level and polymorphism in patients on long-term peritoneal dialysis. Nephrol Dial Transplant. 2005;20:2489-2496. 16115848 10.1093/ndt/gfi089

[87] Meijvis SCA Herpers BL Endeman H et al Mannose-binding lectin (MBL2) and ficolin-2 (FCN2) polymorphisms in patients on peritoneal dialysis with staphylococcal peritonitis. Nephrol Dial Transplant. 2011;26:1042-1045. 20682603 10.1093/ndt/gfq474

[88] Numata M Nakayama M Hosoya T et al Possible pathologic involvement of receptor for advanced glycation end products (RAGE) for development of encapsulating peritoneal sclerosis in Japanese CAPD patients. Clin Nephrol. 2004;62:455-460. 15630905 10.5414/cnp62455

[89] Murea M Lu L Ma L et al Genome-wide association scan for survival on dialysis in African-Americans with type 2 diabetes. Am J Nephrol. 2011;33:502-509. 21546767 10.1159/000327985 PMC3202959

[90] Perl J Davies SJ Lambie M et al The Peritoneal Dialysis Outcomes and Practice Patterns Study (PDOPPS): unifying efforts to inform practice and improve global outcomes in peritoneal dialysis. Perit Dial Int. 2016;36:297-307. 26526049 10.3747/pdi.2014.00288 PMC4881793

