# Supplementary material for: Opportunities and Challenges for Genetic Studies of End-Stage Renal Disease in Canada
Source: Can J Kidney Health Dis. 2018 Jul 22;5:2054358118789368. doi: 10.1177/2054358118789368 (PMC6056781; doi:10.1177/2054358118789368)
Supplement: Supplemental_table_1 – Supplemental material for Opportunities and Challenges for Genetic Studies of End-Stage Renal Disease in Canada [file Supplemental_table_1.pdf]

Supplemental table 1: Association studies relevant to chronic hemodialysis

Problem types	Outcome details	Genes	Sample size	References
Access problem	Graft failure	TNF-a	67	Ram et al.
	Access thrombosis	TGF-b, PAI-1	416	Lazo-Langner et al.
	Access thrombosis	TNF-a, ACE	98	Sener et al.
	AV fistula patency	TGF-b	120	Heine et al.
	AV fistula patency	MMP3, MMP6, MMP9	596	Lin et al.
	Access thrombosis	ACE, MTHFR, CD14	101	Brophy et al.
Biochemical	EPO resistance	IL-1b, ACE	167	Jeong et al.
	Ca PO4	Klotho	118	Marchelek-Myśliwiec et al.
	Secondary hyperparathyroidism	PTH	166	Gohda et al.
	Iron indices	VDR	88	Amato et al.
Cardiovascular morbidity	Cardiovascular morbidity	ENOS	335	Asakimori et al.
	Cardiovascular morbidity	IL-6	775	Liu et al.
	HTN, LVH	IL-6, CX3CR1	161	Losito et al.
	Cardiovascular morbidity	TNF-a, IL-10	167	Yilmaz et al.
	Cardiovascular morbidity	IL-6, IL-10, TNF-a	169	Tosic Dragovic et al.
	Cardiovascular morbidity	HIF-1a	376	Zheng et al.
	Cardiovascular	AGT, AT2R, ACE	160	Losito et al.

	morbidity			
	Cardiovascular morbidity	TGF-b	183	Rao et al.
	HTN	Renalase	137	Kiseljakovic et al.
	Cardiovascular morbidity	IL-10	300	Girndt et al.
	Comorbidity	IL-6, IL-10, TNF-a	183	Balakrishnan et al.
Immunity	Inflammation	IL-6	775	Liu et al.
	Immune function	IL-10	272	Girndt et al.
	Inflammation	INF-g	123	Biolo et al.
Mortality	Mortality	RANTES	225	Böger et al.
	Mortality	VDR	143	Marco et al.
	Mortality	IFNL3, IL12A, IL13, IL4R, CCL2, IL12, IL18	532	Grzegorzewska et al.
	Mortality	VEGF, MMP-1, VDR, CD180, IL-6, TGF-BR1,	1330	Rothuizen et al.
